# Advances on the Acupuncture Therapies and Neuroplasticity

**DOI:** 10.1155/2018/7231378

**Published:** 2018-12-17

**Authors:** Cun-Zhi Liu, Jian-De Chen, Meng Zhang

**Affiliations:** ^1^Department of Acupuncture and Moxibustion, Dongfang Hospital, Beijing University of Chinese Medicine, Beijing, China; ^2^Johns Hopkins School of Medicine, USA; ^3^Northumbria University, UK

Acupuncture as one of complementary therapies is widely used in pain and neurodegenerative diseases in clinical practice. Although many studies have tried to analyze the potential effects of acupuncture, the mechanisms are not fully elucidated yet. As a potent form of sensory peripheral stimulation, acupuncture may affect pain and neurodegenerative diseases via mediating on neural plasticity. Neuroplasticity, including dendritic remodeling, synapse turnover, long-term potentiation (LTP), and neurogenesis, is engaged in development of brain, skills learning, formation and extinction of memory, and self-repair of neural injuries.This special issue collects nine papers concerning acupuncture therapies and neuroplasticity in diverse disciplines (molecular biological technology and imaging technologies).

There are three manuscripts about the neuroprotection of acupuncture on neurologic disease. First, the paper by L. Guo et al. entitled “Electroacupuncture Ameliorates Cognitive Deficit and Improves Hippocampal Synaptic Plasticity in Adult Rat with Neonatal Maternal Separation” suggested that early life stress due to maternal separation may induce adult cognitive deficit associated with hippocampus, and the therapeutic effect of electroacupuncture in young adult may be via ameliorating deficit of hippocampal synaptic plasticity. The study by J. Jiang et al. showed that electroacupuncture treatment could inhibit the inflammation reaction in the hippocampus of Alzheimer's disease animal model. The possible mechanism of electroacupuncture reduced the expression of IL-1*β* and NLRP3 inflammasome relative protein. The paper by D. Lin et al. showed that the acupuncture stimulation on the acupoint (ST-36) could activate the brain-derived neurotropic factor (BDNF) signaling pathways in telomerase deficient mice. This study suggested that the neuroprotection and neuron regeneration maybe play a critical role in electroacupuncture-induced antiaging effect.

The paper by L. Cavalli et al. reported that the beneficial effects of acupuncture on acute severe acquired brain injuries may be related to neuroinflammation, intracranial oedema, oxidative stress, and neuronal regeneration. Acupuncture controlled the imbalance of IGF-1 hormone, decreased spasticity, pain, and the incidence of neurovegetative crisis. The paper by Y.-F. Li et al. indicated that the comprehensive therapy of electroacupuncture in rats with spinal cord injury can effectively enhance the growth of nerve fibers and improve the hindlimb motor function recovery, suggesting that combination therapies could become a powerful treatment for spinal cord injury.

There are two manuscripts using functional magnetic resonance imaging to explore the mechanism underlying acupuncture treatment. The aim of P. Wu et al. study was to observe the grey matter (GM) tissue changes of ischemic stroke in 24 patients. They found acupuncture could evoke pronounced structural reorganization in the frontal areas and the network of DMN areas, which may be the potential mechanism that acupuncture improved the motor and cognition recovery. In the paper by J. Li et al., they used 18F-2-fluorodeoxy-D-glucose positron emission tomography (18F-FDG-PET) to examine the effects of acupuncture at LR3 in spontaneously hypertensive rats. They suggested that acupuncture improves hypertension through a mechanism involving altered brain activation. Acupuncture could decrease the cerebral glucose metabolism in the hypothalamus, thalamus, medulla oblongata, and cerebellum.

In addition, K. Zhang et al. provided an updated state of the effectiveness of acupuncture in obesity by a meta-analysis. They summarized the available studies on exploring the mechanisms under the efficacy of acupuncture in obesity animals and found that acupuncture is an effective treatment for obesity and inferred neuroendocrine regulation might be involved. The study by Z. Ge et al. found home-based transcutaneous neuromodulation improved constipation via modulating gastrointestinal hormones and bile acids

In summary, this issue provides different varies evidences presented by diverse authors covering several topics related to advances in acupuncture for mediating neural plasticity. Neural plasticity could be a bridge between acupuncture and various neurological diseases. More in-depth researches are required to reveal the underlying mechanism of acupuncture.

